# Protective Effects of Caffeic Acid Phenethyl Ester (CAPE) and Novel Cape Analogue as Inducers of Heme Oxygenase-1 in Streptozotocin-Induced Type 1 Diabetic Rats

**DOI:** 10.3390/ijms20102441

**Published:** 2019-05-17

**Authors:** Valeria Sorrenti, Marco Raffaele, Luca Vanella, Rosaria Acquaviva, Loredana Salerno, Valeria Pittalà, Sebastiano Intagliata, Claudia Di Giacomo

**Affiliations:** 1 Department of Drug Science, Biochemistry Section, University of Catania, 95125 Catania, Italy; marco.raffaele@hotmail.com (M.R.); lvanella@unict.it (L.V.); racquavi@unict.it (R.A.); cdigiaco@unict.it (C.D.G.); 2 Department of Drug Science, Pharmaceutical Chemistry Section, University of Catania, 95125 Catania, Italy; lsalerno@unict.it (L.S.); vpittala@unict.it (V.P.); 3 Department of Medicinal Chemistry, College of Pharmacy, University of Florida, Gainesville, FL 32610, USA; s.intagliata@cop.ufl.edu

**Keywords:** Type 1 diabetes mellitus (T1D), Pancreatic oxidative damage, Heme oxygenase-1 (HO-1) inducers, Caffeic acid phenethyl ester (CAPE), Reactive oxygen species (ROS), Dimethylarginine dimethylaminohydrolase-1 (DDAH-1), Inducible nitric oxide synthase (iNOS), Gamma-Glutamyl-Cysteine Ligase (GGCL)

## Abstract

Type 1 diabetes mellitus (T1D) is a chronic autoimmune disease resulting in the destruction of insulin producing β-cells of the pancreas, with consequent insulin deficiency and excessive glucose production. Hyperglycemia results in increased levels of reactive oxygen species (ROS) and nitrogen species (RNS) with consequent oxidative/nitrosative stress and tissue damage. Oxidative damage of the pancreatic tissue may contribute to endothelial dysfunction associated with diabetes. The aim of the present study was to investigate if the potentially protective effects of phenethyl ester of caffeic acid (CAPE), a natural phenolic compound occurring in a variety of plants and derived from honeybee hive propolis, and of a novel CAPE analogue, as heme oxygenase-1 (HO-1) inducers, could reduce pancreatic oxidative damage induced by excessive amount of glucose, affecting the nitric oxide synthase/dimethylarginine dimethylaminohydrolase (NOS/DDAH) pathway in streptozotocin-induced type 1 diabetic rats. Our data demonstrated that inducible nitric oxide synthase/gamma-Glutamyl-cysteine ligase (iNOS/GGCL) and DDAH dysregulation may play a key role in high glucose mediated oxidative stress, whereas HO-1 inducers such as CAPE or its more potent derivatives may be useful in diabetes and other stress-induced pathological conditions.

## 1. Introduction

Diabetes mellitus (DM) is a chronic syndrome of impaired carbohydrate, protein, and fat metabolism caused by insufficient secretion of insulin and/or defects in insulin action in tissues due to insulin resistance. Type 1 diabetes mellitus (T1D) is a chronic autoimmune disease resulting in the destruction of insulin producing β-cells of the pancreas, with consequent insulin deficiency and excessive glucose production [1,2]. Although insulin resistance is traditionally linked to type 2 diabetes mellitus (T2D), intense inflammatory activities characterized by the presence of cytokines, apoptotic cells, immune cell infiltration, amyloid deposits, and fibrosis may result also in T2D due to loss of β-cells and reduced insulin production [3]. Moreover, irrespective of the type, DM is a complex metabolic disease, often associated with long-term complications, including vascular complications, and affecting many tissues [4,5,6,7,8,9]. In the diabetic status, exposure to high levels of glucose cause a marked reduction in endothelial cell (EC)-released NO [10], with consequent vascular dysfunction [11]. Previous studies have shown that endogenous arginine analogs may play a regulatory role in the arginine/NO pathway [12]. Asymmetric NG, NG-dimethyl-l-arginine (ADMA) is an endogenous inhibitor of all isoforms of nitric oxide synthase (NOS). Elevated ADMA levels have been identified as a biomarker of endothelial dysfunction [13], suggesting that plasma ADMA is significantly associated with cardiovascular risk. ADMA metabolism is related to its generation from protein breakdown and to its cleavage by dimethylarginine dimethylaminohydrolase (DDAH) into citrulline and dimethylamine [14]. Two distinct isoforms of DDAH have been described so far, DDAH-1 and DDAH-2, with distinct tissue distribution [15,16]. It has been reported that overproduction of reactive oxygen species (ROS) leads to downregulation of DDAH-1 and -2, as well as ADMA accumulation by inhibiting DDAH enzyme, which can be prevented by antioxidants [17,18]. In most tissues, hyperglycemia results in increased levels of ROS and nitrogen species (RNS). Without adequate compensatory response by endogenous antioxidant systems, a redox imbalance occurs, leading to the activation of specific pathways that can amplify the damage. It has been reported that in diabetic patients the increase in oxidative stress is associated with a decline in cellular antioxidant defenses [7]. The transcription factor called Nrf2 (nuclear factor erythroid-derived 2) is referred to as the “master regulator” of the antioxidant response; it modulates the expression of hundreds of genes, including those with a promoter region containing an antioxidant response element (ARE) [19], such as heme oxygenase-1 (HO-1), DDAH-1, DDAH-2, gamma-Glutamyl-cysteine ligase (GGCL) [20,21,22,23] and other antioxidant/detoxifying enzymes. The pharmacological manipulation of Nrf2 may represent a target in treating metabolic disorders such as diabetes. This research aims to elucidate some biochemical and metabolic aspects of diabetes, identifying any changes in the capacity of antioxidant defense, in an experimental in vivo model of diabetes. In addition, although some experimental data showed unwanted effects of HO-1 induction in diabetic models [24,25,26], as it is also evident that all molecules capable of inducing the biosynthesis of HO-1 may represent potential protective agents, natural compounds and synthetic derivatives of natural molecules could be a valid approach for use as adjuvants in antidiabetic therapy [27]. The phenethyl ester of caffeic acid (CAPE), a natural phenolic compound occurring in a variety of plants and derived from honeybee hive propolis has many beneficial properties (anti-carcinogenic, anti-viral, anti-inflammatory, anti-oxidant) [28,29], however, the mechanisms of pleiotropism of CAPE are not fully understood and are partially attributed to the ability to induce HO-1 expression [30]. Our previous, in vitro, study showed that CAPE and small focused series of CAPE analogues were HO-1 inducers. Some of tested compounds were more potent HO-1 inducers than CAPE.

**Figure 1:**
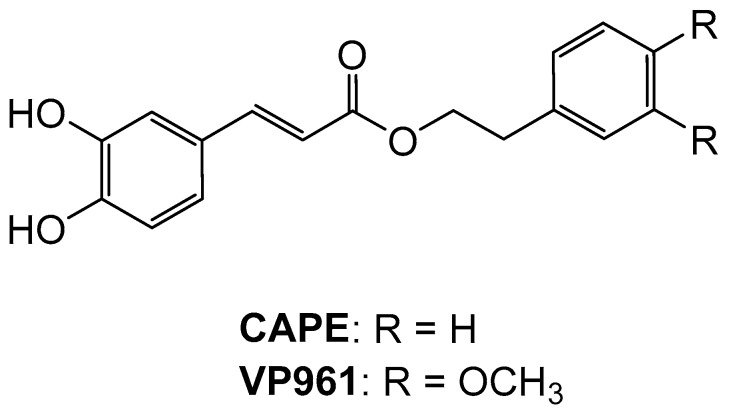
Chemical structure of caffeic acid phenethyl ester (CAPE) and VP961-CAPE derivative.

Particularly, 3-(3,4-dihydroxyphenyl)-(2E)-2-propenoic acid 2-(3,4-dimethoxyphenyl) ethyl ester (VP961) was the most potent (Figure 1). Moreover, VP961 is the first known compound able to directly activate the HO-1 enzyme and to induce its protein expression at the same time [31].

The aim of the present study was to investigate if the potentially protective effect of CAPE as an HO-1 inducer could reduce pancreatic oxidative damage induced by excessive amount of glucose, affecting the NOS/DDAH pathway in streptozotocin-induced type 1 diabetic (STZ) rats. Moreover, because to date only limited strctural CAPE analogues have been examined in vivo [32,33], the protective effect of CAPE derivative VP961, more potent in vitro than the parent compound CAPE as an HO-1 inducer, was investigated in the same animal model mentioned above.

## 2. Results

### 2.1. Body Weight, Blood Glucose Content, Food Intake, Water Intake, and Volume of Urine Excreted

#### 2.1.1. The Effects of CAPE and VP961 on Animal Body Weight

Table 1 shows the time course of the changes in body weight during the experimental period. The diabetic control rats (STZ) displayed a marked decrease in body weight with respect to the normal control rats. Body weight was significantly increased in CAPE- or VP961-treated STZ rats with respect to the diabetic control.

#### 2.1.2. The Effects of CAPE and VP961 on Blood Glucose Content

**Figure 2:**
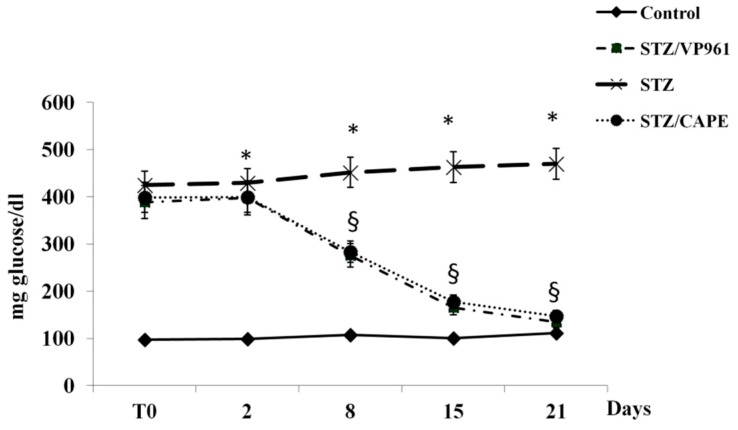
Effects of CAPE and VP961 on blood glucose content during the experimental period. Values are mean ± standard deviation (S.D.) of three independent experiments performed in triplicate. * *p* < 0.05 vs. normal control rats; ^§^ *p* < 0.05 vs. diabetic control rats (STZ).

Figure 2 shows the time course of the changes in blood glucose content during the experimental period. After two days, a significant increase was observed in both diabetic control rats (STZ) and in CAPE- or VP961-treated STZ rats with respect to normal control rats. A significant reduction in blood glucose content was observed in CAPE- or VP961-treated STZ rats with respect to diabetic control rats after 8, 15, and 21 days of treatment.

#### 2.1.3. The Effects of CAPE and VP961 on Water Intake, Volume of Urine Excreted, and Food Intake

**Figure 3:**
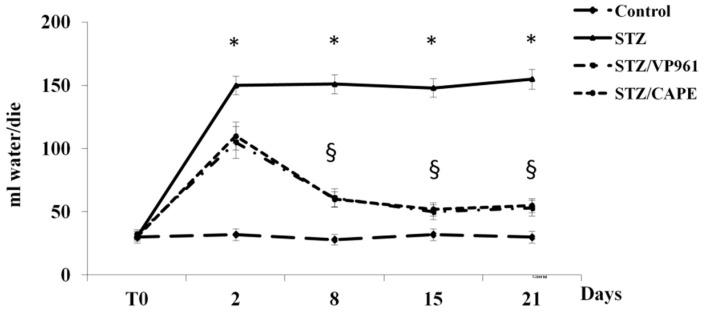
Effects of CAPE and VP961 on water intake during the experimental period. Values are mean ± standard deviation (S.D.) of three independent experiments performed in triplicate. * *p* < 0.05 vs. normal control rats; ^§^ *p* < 0.05 vs. diabetic control rats (STZ).

**Figure 4:**
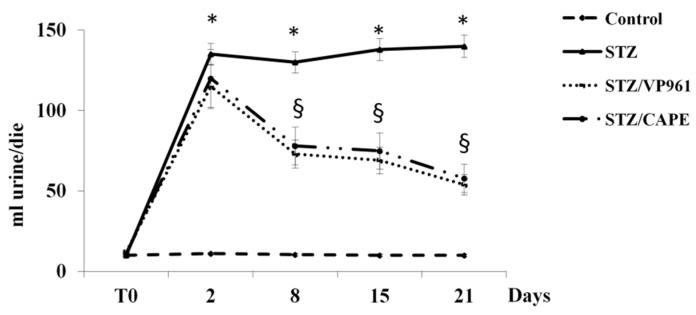
Effects of CAPE and VP961 on volume of urine excreted during the experimental period. Values are mean ± standard deviation (S.D.) of three independent experiments performed in triplicate. * *p* < 0.05 vs. normal control rats; ^§^ *p* < 0.05 vs. diabetic control rats (STZ).

The time course of the changes in water intake and volume of urine excreted during the experimental period shows that after two days, a significant increase was observed in both diabetic control rats and in CAPE- or VP961-treated STZ rats with respect to normal control rats. A significant reduction in water intake and volume of urine excreted was observed in CAPE- or VP961-treated STZ rats with respect to diabetic control rats (STZ) after 8, 15, and 21 days of treatment (Figure 3 and Figure 4). 

The food intake of normal rats was higher with respect to STZ rats (normal control = 25 ± 2 g/day; diabetic control rats (STZ) = 35 ± 3 g/day). The food intake of STZ rats treated with CAPE or VP961 was similar to that of normal rats. 

### 2.2. Plasma Insulin, RSH, LOOH, ADMA, and Nitrite/Nitrate Levels 

As shown in Table 2, the plasmatic insulin and non-proteic thiol groups (RSH) levels were significantly lower in the diabetic control rats (STZ) than that in the non-STZ rats. Treatment with CAPE or VP961 significantly increased these levels. The levels of lipid hydroperoxide (LOOH), an oxidative stress biomarker, in the plasma of diabetic control rats (STZ) were significantly elevated compared with non-STZ rats; however, these levels were significantly decreased upon receiving CAPE or VP961. STZ rats had increased plasmatic ADMA and NO_2_^−^/NO_3_^−^ levels compared to the normal control group. CAPE or VP961 treatment in STZ rats significantly reduced ADMA and NO_2_^−^/NO_3_^−^ levels with respect to control STZ rats.

### 2.3. Pancreatic RSH, LOOH, ADMA, and Nitrite/Nitrate Levels 

Concerning the pancreatic RSH content, the diabetic control rats (STZ) showed a marked decrease compared with non-diabetic control rats. This content was significantly increased by CAPE or VP961 treatment, as shown in Table 3. The levels of pancreatic LOOH, an oxidative stress biomarker, in diabetic control rats (STZ) were significantly elevated compared with non-STZ rats; however, these levels were significantly decreased upon receiving CAPE or VP961. STZ rats had increased pancreatic ADMA and NO_2_^−^/NO_3_^−^ levels compared to the normal control group. CAPE or VP961 treatment in STZ rats significantly reduced ADMA and NO_2_^−^/NO_3_^−^ levels respect to control STZ rats (Table 3).

### 2.4. Pancreatic HO-1, DDAH-1, GGCL, iNOS Protein Expressions

**Figure 5:**
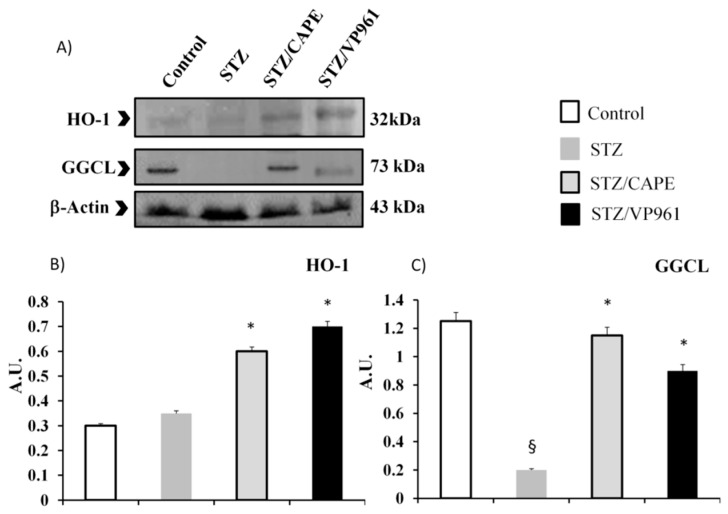
Representative Western blotting of HO-1 and GGCL protein expressions (Panel **A**). Densitometric quantification of HO-1 and GGCL protein expressions in the pancreas of non-STZ rats (control), STZ rats, and CAPE- or VP961-treated STZ rats (CAPE/STZ; VP961/STZ) (Panel **B**,**C**). Values are mean ± standard deviation (S.D.) of three independent experiments performed in triplicate. * *p* < 0.05 vs. diabetic control rats (STZ); ^§^ *p* < 0.05 vs. normal control rats.

The expression levels of antioxidant enzyme-related proteins, such as HO-1 and GGCL, in diabetic control rats (STZ) were very low (Figure 5, Panels B-C). In more detail, HO-1 protein was weakly expressed both in STZ rats and in non-STZ rats, however, CAPE or VP961 administration in STZ rats resulted in a significant upregulation (Figure 5, Panel B).

The expression levels of GGCL in STZ rats were significantly lower than those of non-STZ rats, as shown in Figure 5 (Panel C). The decreased protein expression of GGCL in STZ rats was increased by CAPE or VP961 administration. 

**Figure 6:**
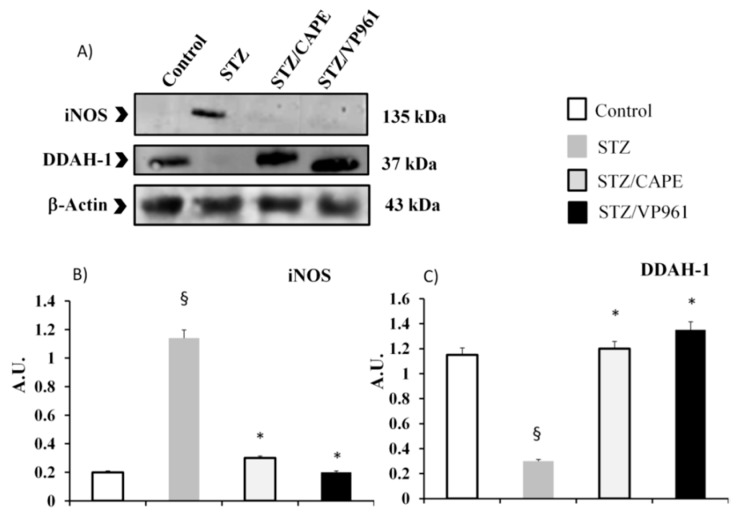
Representative Western blotting of iNOS and DDAH-1 protein expressions (Panel **A**). Densitometric quantification of iNOS and DDAH-1 protein expressions in the pancreas of non-STZ rats (control), STZ rats, and CAPE- or VP961-treated STZ rats (CAPE/STZ; VP961/STZ) (Panel **B**,**C**). Values are mean ± standard deviation (S.D.) of three independent experiments performed in triplicate. * *p* < 0.05 vs. diabetic control rats (STZ); ^§^ *p* < 0.05 vs. normal control rats.

The expression levels of iNOS protein in diabetic control rats were significantly higher than those of non-STZ rats, as shown in Figure 6 (Panel B). The increased protein expression of iNOS in STZ rats was decreased by CAPE or VP961 administration. 

The expression levels of DDAH-1 protein in diabetic control rats were significantly lower than those of non-STZ rats, as shown in Figure 6 (Panel C). The decreased protein expression of DDAH-1 in STZ rats was increased by CAPE or VP961 administration. 

## 3. Discussion

ROS and RNS are well recognized for playing a dual role in human pathology as both deleterious and beneficial species [34]. In addition, it is often difficult to distinguish whether oxidative reactions occurring during a disease process are the cause, by participating in the initial pathogenetic mechanisms of tissue damage, or if they appear only as one of the final effects of the process [35]. Attempts have been made to reduce oxidative damage related to diabetes complications, but the results of administration of antioxidants were disappointing [36,37]; for these reasons, currently research is aiming at the identification of so-called “indirect antioxidants,” and the stimulation and strengthening of endogenous antioxidant defenses [38,39]. In recent years, much attention has been focused on phyto-constituents present in fruits, vegetables, and medicinal herbs, and, in particular, on some plant secondary metabolites such as phenolic and terpene compounds [40]. There are now many published studies on their antioxidant activities or their ability to enhance endogenous antioxidant defenses by modulating the cellular redox state of plant-derived substances [41,42,43,44,45,46,47,48], but their potential beneficial effects on human health are not confined to their antioxidant action; in fact, numerous interesting biological activities could reveal new roles of these compounds in the prevention and treatment of certain diseases, such as metabolic syndrome and/or diabetes complications [37,49,50]. However, it is important to note that most studies were conducted using cell models, while few results were obtained using in vivo models [36,37]. Type 1 diabetes leads to high blood glucose levels (hyperglycemia) that can cause serious health complications [51]. Although hyperglycemic damage is a multifactorial process, data in the literature suggest that oxidative/nitrosative stress and stress-activated signaling pathways might represent a unifying hypothesis [7]. In diabetic patients, the overproduction of ROS and RNS is associated with iNOS overexpression, which might contribute to stress-induced pancreatic cell death [52,53]. 

In our experimental conditions, the significant increase of plasmatic and pancreatic LOOH and nitrite/nitrate levels, markers of oxidative/nitrosative stress, induced by low insulin content and consequent hyperglycemia, may be related to upregulation of pancreatic iNOS protein.

Moreover, oxidative stress is also related to depletion of antioxidant defenses, which also contributes to many of the complications of diabetes, including vascular complications. It has been reported that the overproduction of free radicals could cause damage and apoptosis of pancreatic islet β-cells and reduction of insulin secretion [54]. Bruce et al. reported that HO-1 mRNA expression is significantly reduced in T2D patients [55], whereas upregulation of the HO system increases pancreatic β-cell insulin release and reduces hyperglycemia in different diabetic models [56]. In vitro and in vivo studies have demonstrated that CAPE has many beneficial properties, including anti-hyperglycemic and antioxidant properties [57,58,59,60,61]. In our experimental conditions, body weight of CAPE- or VP961-treated STZ rats was significantly increased compared to the diabetic control rats. Moreover, treatment of STZ rats with CAPE or VP961 significantly reduced blood glucose levels, increased plasmatic insulin levels, and decreased plasmatic and pancreatic LOOH and nitrite/nitrate levels with respect to control STZ rats. The reduction of plasmatic and pancreatic nitrite/nitrate levels may be related to iNOS downregulation in CAPE- or VP961-treated STZ rats. These results suggest that the effects of CAPE or VP961 may be due to their protecting the pancreatic tissue from damage. Moreover, the significant increase of plasmatic and pancreatic LOOH induced by low insulin content and consequent hyperglycemia may be related to downregulation of pancreatic antioxidant defenses, both enzymatic and nonenzymatic, such as HO-1, GGCL, and RSH. Under physiological conditions, Nrf2 locates in the cytoplasm and binds to its inhibitor, kelch-like ECH associated protein 1 (KEAP1). Upon exposure of cells to natural phenolic compounds, Nrf2 is freed from KEAP1 and translocates into the nucleus to bind to antioxidant-responsive elements (ARE) in the genes encoding antioxidant enzymes such as heme oxygenase-1 (HO-1) DDAH-1, DDAH-2, gamma-Glutamyl-cysteine ligase (GGCL) [20,21,22], and other antioxidant/detoxifying enzymes. Our experimental data showed that HO-1 protein at basal levels was weakly expressed in the pancreatic tissue of control rats. These data are in agreement with the data of Li et al. [62]. Since HO-1 protein was also weakly expressed in the pancreatic tissue of STZ rats, any downregulation could not be detectable. However, the levels of HO-1 protein were significantly increased by CAPE or VP961 administration. According to Ye et al. [63], HO-1 induction may be protective of pancreatic β-cells because of the scavenging of free heme, the antioxidant effects of the end product bilirubin, or the generation of carbon monoxide, which might have insulin-secretion-promoting effects and inhibitory effects on nitric oxide synthase. Moreover, VP961 in vivo was, slightly but significantly, more potent than CAPE as an HO-1 inducer. In our experimental conditions in pancreas, GGCL protein, which catalyzes the first and also limiting step in the synthesis of the antioxidant glutathione (GSH), was downregulated in STZ rats, but the levels of this protein were significantly increased by CAPE or VP961 administration. The increased expressions of GGCL induced in STZ rats treated with CAPE or VP961 are related to increased levels of plasmatic and pancreatic RSH and to decreased levels of plasmatic and pancreatic LOOH. Our results demonstrate that in vivo CAPE is more potent than VP961 as a GGCL inducer. 

It has been reported that overproduction of ROS leads to downregulation of DDAH-1 and -2, as well as ADMA accumulation, by inhibiting the DDAH enzyme, which can be prevented by antioxidants [17,18]. Numerous experimental data have shown that DDAH activities are crucial in the regulation of ADMA metabolism [64,65,66] and in the prevention of endothelial dysfunction. Newsholme et al. reported that oxidative stress and ADMA accumulation could lead to pancreatic β-cell dysfunction and decreased insulin secretion, thus compounding the problematic metabolic status of diabetes [67]. In our experimental conditions, DDAH-1 protein, the main isoform of DDAH expressed in pancreas, was also downregulated in STZ rats, but the levels of this protein were significantly increased by CAPE or VP961 administration. The increased expression of DDAH-1 induced in STZ rats treated with CAPE or VP961 may be due to Nrf2 translocation into the nucleus and to its binding to antioxidant-responsive elements (ARE) in the genes encoding DDAH-1. DDAH-1 upregulation is related to decreased levels of plasmatic and pancreatic ADMA. Our results demonstrate that VP961 was more potent in vivo than CAPE as a DDAH-1 inducer. Overall, our data demonstrated that in an animal model of T1D, CAPE or VP961 treatment may reverse the diabetes-induced oxidative stress in rat pancreas.

## 4. Materials and Methods 

### 4.1. Animal Model

All animal procedures were performed in accordance with the Guidelines for Care and Use of Laboratory Animals of “Catania University,” and experiments were approved by the Animal Ethics Committee (project code N.170, Italy; 1 October 2016) of “MINISTRY OF HEALTH (Directorate General for Animal Health and Veterinary Medicines) (Italy)”. Thirty day old Wistar rats were purchased from Charles River Labs (Lecco, Italy). The rats were maintained under a 12 h light/dark cycle, and housed in a controlled temperature (24 ± 2 °C) and humidity (50 ± 5%) environment. After several days of adaptation, the rats were divided into normal and diabetic groups. The experimental diabetes was induced by intraperitoneal (i.p.) injection of streptozotocin (50 mg/kg body weight in a 10 mM citrate buffer, pH 4.5). One week after the injection, we verified the occurrence of hyperglycemia; animals with blood glucose >140 mg/dL were placed in individual metabolic cages; body weight, amount of water and food taken, and volume of urine excreted were recorded daily. Non-fasting blood samples were collected twice per week by tail bleeding into heparinized tubes. In the plasma samples, the glucose concentrations were determined. Rats were distributed in four groups: group I included six untreated animals that were considered the normal control group; group II included six diabetic animals considered the diabetic control group; group III included six diabetic animals orally treated with a non-toxic dose (30 mg/Kg) of the alcoholic extract of CAPE; and group IV included six diabetic animals orally treated with a non-toxic dose (30 mg/Kg) of the alcoholic extract of CAPE derivative VP961. Control groups (diabetic and non-diabetic rats) received the same volume of ethanol as vehicle.

After 21 days, animals were sacrificed by an overdose of anesthetic, and blood and pancreas tissues were immediately removed and frozen for biochemical assays. 

### 4.2. Measurement of Glucose and Insulin in the Plasma

Plasmatic glucose and insulin levels were measured using, respectively, a commercial glucose ELISA kit (CrystalChem, Zaandam, the Netherlands) and commercial insulin ELISA kit (ALPCO, Salem, NH, USA) in accordance with the manufacturer’s instructions. Results are reported respectively as mg glucose/dl of plasma and ng insulin/mL of plasma.

### 4.3. Plasmatic and Pancreatic Nitrite/Nitrate Determination

Quantification of nitrite, the stable metabolite of nitric oxide, was measured colorimetrically via Griess reaction. Aliquots of plasma or pancreas homogenates were preincubated for 30 min at room temperature with 50 μM nicotinamide adenine dinucleotide phosphate (Sigma-Aldrich, St. Louis, MO, USA) and 24 mU nitrate reductase (Roche Diagnostics Gmbh, Mannheim, Germany), and then the samples were treated with 0.2 U lactate dehydrogenase (Roche) and 0.5 mol sodium pyruvate for 10 min. The coloration was developed by adding Griess reagent (Merck KGaA, Darmstadt, Germany; 1:1, vol/vol). Finally, after 10 min at room temperature, absorbance was recorded by 96 well plate microtiter at λ 540 nm. Nitrite levels were determined using a standard curve and expressed as nanomoles of NO_2_^−^/NO_3_^−^ per ml of plasma or NO_2_^−^/NO_3_^−^ per milligram of protein. Protein concentration was measured using TAKE 3 nanodrop.

### 4.4. Plasmatic and Pancreatic ADMA Determination

Plasma and tissue ADMA concentration was determined in plasma or pancreas homogenates using a commercially available enzyme-linked immunosorbent assay kit (DLD Diagnostika GmbH, Hamburg, Germany) according to the manufacturer’s instructions. Results are reported as nmoles ADMA/mL of plasma or nmoles ADMA/mg prot.

### 4.5. Determination of Plasmatic and Pancreatic Lipid Hydroperoxide Levels

Plasma and pancreatic levels of lipid hydroperoxide were evaluated following the oxidation of Fe^+2^ to Fe^+3^ in the presence of xylenol orange at λ 560 nm, as previously described [68]. Results are reported as nmoles LOOH/mL of plasma or nmoles LOOH/mg prot.

### 4.6. Non-Proteic Thiol Groups Determination

Plasma and pancreatic levels of non-proteic thiol groups were measured, in 200 μL of plasma or pancreatic homogenate, using a spectrophotometric assay, as previously described [68]. Results are reported as nmoles RSH/mL of plasma or nmoles RSH/mg prot.

### 4.7. Western Blotting

Western blotting analysis was performed as previously described [69,70]. Briefly, tissues were homogenized in lysis buffer (50 mM Tris-HCl, 10 mM EDTA, 1% *v*/*v* Triton X-100, 1% phenylmethylsulfonyl fluoride (PMSF), 0.05 mM pepstatin A, and 0.2 mM leupeptin) and tissue homogenates (30 μg proteins) were loaded onto 12% SDS-polyacrylamide (SDS-PAGE) gels and subjected to electrophoresis (120 V, 90 min). The separated proteins were transferred to nitrocellulose membranes (Bio-Rad, Hercules, CA, USA). After transfer, the blots were incubated with Li-COR blocking buffer for 1 h, followed by overnight incubation with primary antibodies directed against HO-1 (1:1000) [Enzo Life Sciences, Plymouth Meeting, PA)], GGCL (1:1000) [Abcam, Cambridge, United Kingdom], DDAH-1 (1:5000) [Calbiochem EMD Biosciences (Darmstadt, Germany)], iNOS (1:1000) (SantaCruz Biotechnology, Santa Cruz, CA, USA) and β-actin (Cell Signaling Technology, Inc., Danvers, MA, USA). After washing with TBS, the blots were incubated for 1 h with the secondary antibody (1:1000). Protein detection was carried out using a secondary infrared fluorescent dye-conjugated antibody absorbing at λ 800 and λ 700 nm. The blots were visualized using an Odyssey Infrared imaging scanner (LI-COR Biosciences), and quantified by densitometric analysis performed after normalization with β-actin. Results are expressed as arbitrary units (A.U.).

### 4.8. Statistical Analysis

Data are reported as mean ± standard deviation (S.D.) values of at least three independent experiments. The results were analyzed for statistical significance using ANOVA, followed by Bonferroni’s post hoc test. A *p*-value < 0.05 was considered as significant.

## 5. Conclusions

Overproduction and/or insufficient removal of free radicals results in different pathological conditions, including diabetes [71]. Diabetes mellitus increases oxidative stress in pancreatic tissue. Oxidative damage of the pancreatic tissue may contribute to endothelial dysfunction associated with diabetes. It can be concluded that CAPE and VP961 inhibit lipid peroxidation and regulate antioxidant enzyme-related proteins in STZ rats. Moreover, iNOS/GGCL and DDAH dysregulation may play a key role in high glucose mediated oxidative stress, whereas HO-1 inducers such as CAPE or its derivatives may be useful in treating diabetes and other stress-induced pathological conditions.

## Figures and Tables

**Table 1:** Effects of CAPE and VP961 on body weight during the experimental period.

Groups	T0	8 Days	15 Days	21 Days
	Body Weight (g)	Body Weight (g)	Body Weight (g)	Body Weight (g)
Control	231 ± 3	265 ± 5	300 ± 7	335 ± 11
STZ	228 ± 5	238 ± 7*	262 ± 5 *	280 ± 3 *
STZ/CAPE	220 ± 3	256 ± 9	291 ± 3	329 ± 4
STZ/VP961	226 ± 5	253 ± 8	291 ± 4	318 ± 6

Values are mean ± standard deviation (S.D.) of three independent experiments performed in triplicate. * *p* < 0.05 vs. normal control rats.

**Table 2:** Plasmatic insulin, non-proteic thiol groups, lipid hydroperoxide, Asymmetric NG, NG-dimethyl-l-arginine (RSH, LOOH, ADMA), and NO_2_^−^/NO_3_^−^ levels.

PLASMA	Insulin (ng/mL)	RSH (nmoles/mL)	LOOH (nmoles/mL)	ADMA (nmoles/mL)	NO_2_^−^/NO_3_^−^ (nmoles/mL)
Control	1.0 ± 0.03	140 ± 10	15 ± 2	0.1 ± 0.02	0.75 ± 0.03
STZ	0.45 ± 0.05 *	80 ± 7 *	30 ± 5 *	0.9 ± 0.03 *	1.5 ± 0.05 *
STZ/CAPE	0.82 ± 0.03 **	130 ± 9 **	18 ± 3 **	0.6 ± 0.02 **	0.8 ± 0.03 **
STZ/VP961	0.78 ± 0.07 **	120 ± 8 **	17 ± 4 **	0.3 ± 0.02 **±	0.7 ± 0.04 **

Values are mean ± standard deviation (S.D.) of three independent experiments performed in triplicate. * *p* < 0.05 vs. normal control rats; ** *p* < 0.05 vs. diabetic control rats (STZ).

**Table 3:** Pancreatic RSH, LOOH, ADMA, and NO_2_^−^/NO_3_^−^ levels.

PANCREAS	RSH (nmoles/mg prot.)	LOOH (nmoles/mg prot.)	ADMA (nmoles/mg prot.)	NO_2_^−^/NO_3_^−^ (nmoles/mg prot.)
Control	28 ± 2	0.2 ± 0.03	20 ± 0.8	4 ± 0.9
STZ	12 ± 1 *	1 ± 0.04 *	200 ± 5 *	12 ± 2 *
STZ/CAPE	27 ± 2 **	0.4 ± 0.02 **	50 ± 4 **	6 ± 0.8 **
STZ/VP961	26 ± 3 **	0.3 ± 0.03 **	53 ± 3 **	5 ± 0.9 **

Values are mean ± standard deviation (S.D.) of three independent experiments performed in triplicate. * *p* < 0.05 vs. normal control rats; ** *p* < 0.05 vs. diabetic control rats (STZ).
